# Office based non-invasive diagnostic technique for acquired tracheoesophageal fistula

**DOI:** 10.1016/j.bjorl.2024.101466

**Published:** 2024-07-10

**Authors:** Nesha Rajendram, Masaany Mansor, Norazila Abdul Rahim, Mohd Zukiflee Abu Bakar, Muhammad Arif Sobani

**Affiliations:** a University Malaya, Faculty of Medicine, Department of Otorhinolaryngology Head and Neck Surgery, Kuala Lumpur, Wilayah Persekutuan Kuala Lumpur, Malaysia; b Universiti Teknologi MARA (UiTM), Faculty of Medicine, Department of Otorhinolaryngology Head and Neck Surgery, Sungai Buloh, Selangor, Malaysia; c Hospital Al-Sultan Abdullah UiTM, Department of Otorhinolaryngology, Bandar Puncak Alam, Selangor, Malaysia

## Introduction

Fistulisation between the airway and the gastrointestinal tract is an uncommon problem that results from a spectrum of disease processes. Etiology of acquired tracheoesophageal fistula includes penetrating trauma, malignancies, foreign bodies in trachea or esophagus, granulomatous infection, iatrogenic injuries post intubation, surgeries following esophagectomy, laryngectomy or tracheostomy.^1^ Regardless of its cause, the disastrous pulmonary sepsis due to its ongoing tracheobronchial contamination, and the interference with nutrition are all lethal facets of this disease. Due to its rarity, subtle clinical presentations, and invasive diagnostic test, Tracheoesophageal Fistulas (TOF) is often missed, or the diagnosis and treatment delayed. Hence, we report our invention with regards of a minimally invasive, technique in diagnosing TOF in an office-based setting.

## Case report

A 77-year-old man with ECOG status 2 was tracheostomised 3-years ago due to prolonged intubation after a cardiogenic shock. He was then discharged post tracheostomy 1 month later with Shiley tube. However, he defaulted follow up until 10 months post tracheostomy when he complained of hoarseness. Upon assessment he was noted to have well compensated right vocal fold palsy. Fibreoptic Endoscopic Evaluation Swallowing (FEES) examination was done showed no risk of aspiration therefore he was decannulated.

During his subsequent follow ups, 2-years post decannulation, he was noted to have subglottic stenosis Cotton Mayer Grade II and referred to our Laryngology clinic. His subglottic stenosis progressed to Cotton Mayer 3, and he required a re-tracheostomy under local anaesthesia, complicated by oesophageal injury whereby a TOF was suspected as patient noted food particles from tracheostomy site immediately post op re-tracheostomy upon feeding was commenced. Feeding via nasogastric tube was immediately commenced. He did not experience cough episodes after eating, weight loss or developed recurrent aspiration pneumonia as a result of the tracheoesophageal fistula.

Conventionally to localize a TOF, it involved direct visualisation under General Anaesthesia (GA). Due to his multiple comorbidities, this patient was not fit for GA. Subsequently we attempted the nasogastric tube methylene blue technique in clinic in order to locate, diagnose and assess the tracheoesophageal fistula. This method is useful for patients prone for aspiration since the methylene blue can be passed down through the nasogastric tube itself and not via as per oral.

**Figure 1:**
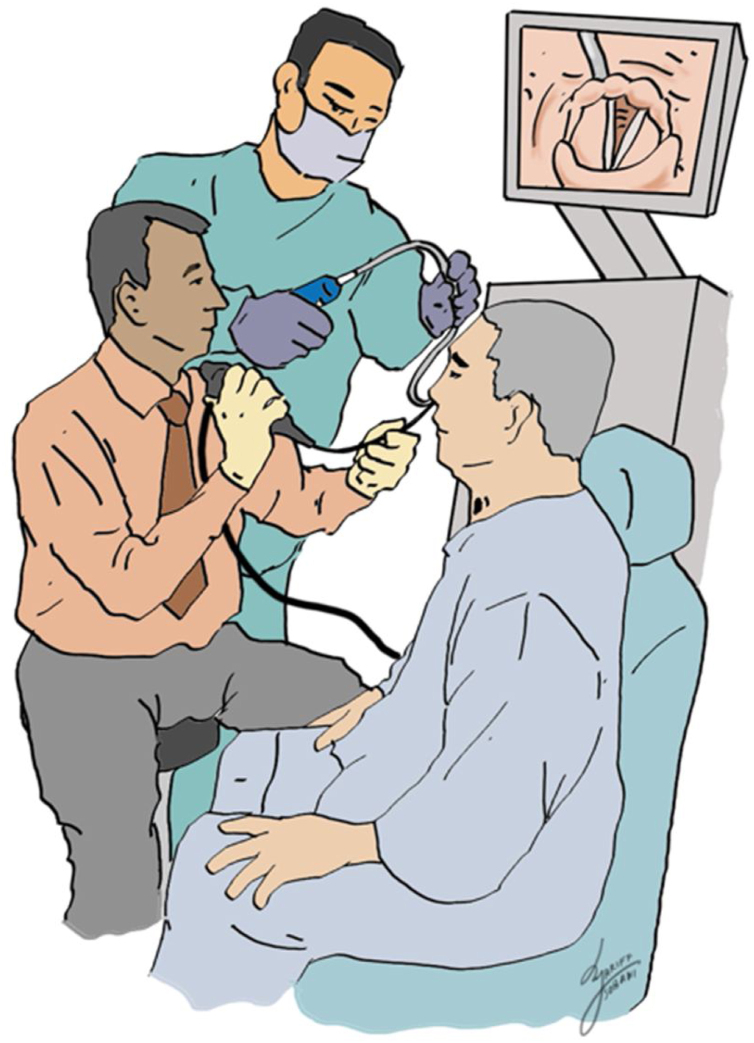
The nasogastric tube is slowly withdrawn until only the tip remains just under the hypopharynx nasopharyngolaryngoscope assisted.

**Figure 2:**
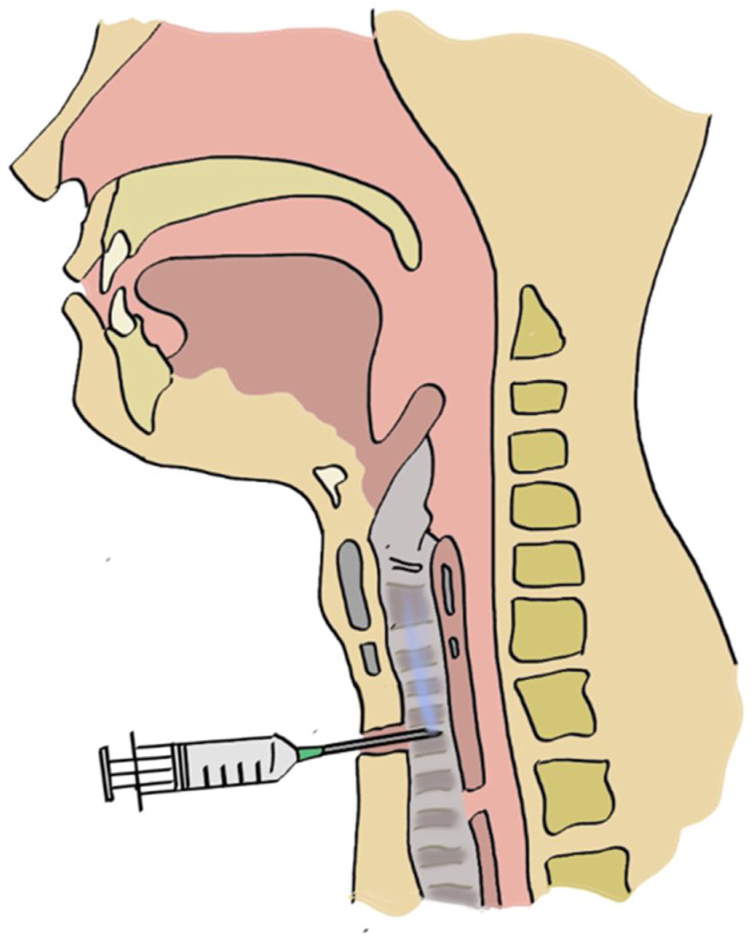
Local anaesthesia using 4 mls of 2% lignocaine or 1‒2 sprays of 10% lignocaine is applied through the tracheostoma with the end needle/nozzle bevelled up to anaesthetize the airway.

**Figure 3:**
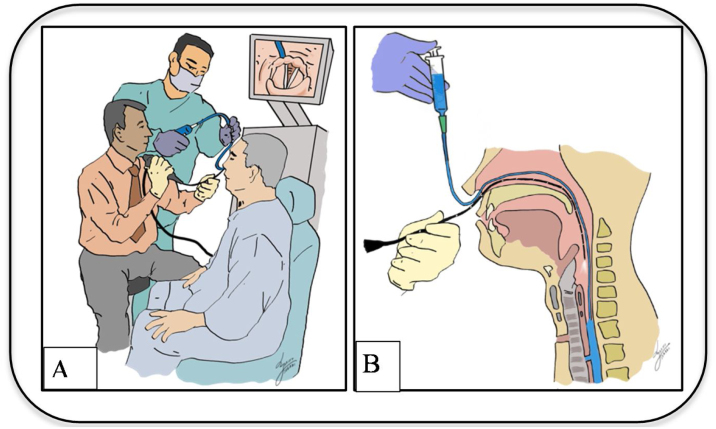
(A‒B) 20 ml of saline mixed with methylene blue is then passed through the nasogastric tube slowly at the speed of 2 ml/10 s to ensure there will be no spillage of the dye around the hypopharyngeal and the laryngeal structures aided by flexible nasopharyngolaryngoscopy.

**Figure 4:**
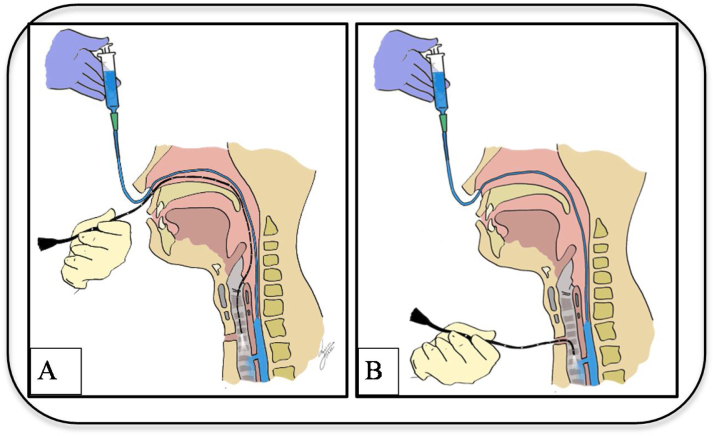
(A‒B) The flexible nasolaryngoscope is then passed through the nostrils & tracheostoma site and manoeuvred to examine the subglottic and trachea region, to visualise for any dye leak which could demarcate clearly any TOF.

Patient in the sitting position, whereby the physician is standing in front of patient. With the nasogastric tube in-situ and functioning, an Olympus brand flexible nasopharyngolaryngoscope size 5 mm connected to Olympus monitor system is introduced until the laryngeal structures. The nasogastric tube is slowly withdrawn until only the tip remains just under the hypopharynx nasopharyngolaryngoscope assisted (Fig. 1). Local anaesthesia using 4 ml of 2% lignocaine or 1‒2 sprays of 10% lignocaine is applied through the tracheostoma with the end needle/nozzle bevelled up to anaesthetize the airway and enable a detailed tracheoscopy to be performed on the patient later (Fig. 2). 20 ml of saline mixed with methylene blue is then passed through the nasogastric tube slowly at the speed of 2 ml/10 s to ensure there will be no spillage of the dye around the hypopharyngeal and the laryngeal structures endoscopy aided (Fig. 3A and B). Subsequently, the flexible nasolaryngoscope is then passed through the nostrils & tracheostoma site and manoeuvred to examine the subglottic and trachea region, to visualise for any dye leak which could demarcate clearly any TOF (Fig. 4A and B). Tracheoesophageal perforation site was visualised at 4th tracheal ring level.

## Discussion

Acquired Tracheoesophageal Fistula (TOF) though rare continues to be a challenging condition to diagnose and manage. It can be due to a variety of causes listed above. In our case, the tracheoesophageal fistula most likely occurred during his re-tracheostomy under local anaesthesia.

Clinical presentations of TOF may range from severe uncontrolled coughing on swallowing (Ono’s sign), acute dysphagia, shortness of breath, hoarseness, pneumonia, chest pain, pyrexia of unknown origin, haemoptysis, and aspiration. In critical cases, patients can present with severe respiratory distress.^2^ In sedated and ventilated patients, TOF should be suspected if a continued air-leak in the ventilator circuit is detected despite a well-inflated cuff. Other signs, include abdominal bloating with ongoing ventilation, loss of tidal volume, worsening oxygenation, recurrent pulmonary sepsis and repeated failures to wean off ventilator.^3^

Presence of oesophageal dilatation with air distal to the fistula of plain chest radiographs is pathognomonic for TOF. However, it is rarely seen. In addition, plain radiography might show the cuff of the tracheostomy tube appearing outside of the tracheal lumen.^1^ In these patients, Computed Tomography (CT) scan of the chest can be performed to evaluate for signs of fistula, aerodigestive tract anatomy and mediastinal pathology. There are no available data assessing the sensitivity, specificity, negative or positive predictive value of CT scans in diagnosing TOF.^3^ Contrast swallow is a highly sensitive and specific modality to detect lesions in the pharynx and oesophagus, in patients who are cooperative for this test. Barium swallow is not advisable as leakage of barium may result in pneumonitis and mediastinitis.^4^ On the other hand, both the digestive tract and the trachea can be assessed by means of endoscopy which is usually performed in the operating room under general anaesthesia.^4^ It is ideal for patients who cannot undergo contrast studies.

However, concerns arise when patients are at high risk or unable to undergo general anaesthesia, and in cases of confirmed tracheoesophageal fistula follow up assessments. With regards to our patient, he was not fit for general anaesthesia due to his underlying multiple comorbidities and cardiac status. Therefore, this non-invasive office-based technique was invented, whereby our main aim was to detect a tracheoesophageal fistula without subjecting patient to general anaesthesia. With the use of a flexible nasoendoscopy set, a nasogastric tube, methylene blue dye and lignocaine spray, the procedure can be done at any clinic-based setting. Within the field of otorhinolaryngology, methylene blue is frequently administered orally in different procedures such as FEES, Evans blue dye test to identify aspiration in tracheostomized patients, and anastomotic leakages following head and neck cancer surgeries.^5^ Hence, due to its rare complications or adverse effects, methylene blue was used in our innovation method. Apart from not exposing patients to the risks of general anaesthesia, to diagnose TOF, to examine post op TOF repair patients, this technique can easily be conducted to assess and monitor the recovery of a TOF. Early diagnosis, leading to early intervention could prevent patients from TOF’s dangerous potentially lethal complications.

## Conclusion

Tracheoesophageal Fistula (TOF) although rare has potentially fatal complications and is difficult to diagnose. The nasogastric tube methylene blue technique we describe above spares patients from general anaesthesia. It is a simple office-based procedure that can diagnose TOF accurately. This non-invasive technique is practical, utilizes instruments in the clinic itself, is comfortable to the patient, and inexpensive.

## Patient consent

Written informed consent was obtained from the patient for the publication of this case report.

## Ethics

Our institution does not require ethical approval for reporting individual cases or case series. In the context of reporting individual cases or case series, our institution has a policy in place that does not mandate the submission of formal ethical approval, recognizing the observational and often retrospective nature of these studies, the minimal risks posed to participants, the emphasis on preserving patient privacy, and the limited generalizability of findings to broader populations.

## Funding

This research received no specific grant from any funding agency in the public, commercial, or not-for-profit sectors.

## Conflicts of interest

The authors declare no conflicts of interest.
